# Formation of Nickel Oxide Nanocuboids in Ferromagnetic La_2_Ni_1−x_Mn_1+x_O_6_

**DOI:** 10.3390/nano11030804

**Published:** 2021-03-21

**Authors:** Monica Bernal-Salamanca, Zorica Konstantinović, Carlos Frontera, Víctor Fuentes, Alberto Pomar, Lluis Balcells, Benjamín Martínez

**Affiliations:** 1 Institut de Ciència de Materials de Barcelona-Consejo Superior de Investigaciones Científicas (ICMAB-CSIC), Campus de Universitat Autonoma de Barcelona, 08193 Bellaterra, Spain; frontera@icmab.es (C.F.); vfuentes@icmab.es (V.F.); apomar@icmab.es (A.P.); benjamin@icmab.es (B.M.); 2 Center for Solid State Physics and New Materials, Institute of Physics Belgrade, University of Belgrade, Pregrevica 118, 11080 Belgrade, Serbia

**Keywords:** ferromagnetic double perovskite, spontaneous formation of NiO_x_ nanocuboids, local transport properties

## Abstract

The control of the spontaneous formation of nanostructures at the surface of thin films is of strong interest in many different fields, from catalysts to microelectronics, because surface and interfacial properties may be substantially enhanced. Here, we analyze the formation of nickel oxide nanocuboids on top of La_2_Ni_1−x_Mn_1+x_O_6_ double perovskite ferromagnetic thin films, epitaxially grown on SrTiO_3_ (001) substrates by radio-frequency (RF) magnetron sputtering. We show that, by annealing the films at high temperature under high oxygen partial pressure, the spontaneous segregation of nanocuboids is enhanced. The evolution of the structural and magnetic properties of the films is studied as a function of the annealing treatments at different temperatures. It is shown that the formation of NiOx nanocuboids leads to a nanostructured film surface with regions of locally different electrical transport characteristics.

## 1. Introduction

Magnetic insulators have gained renewed interest due to their promising properties as material platforms with efficient magnetization dynamics [1,2]. In this regard, double perovskite La_2_NiMnO_6_ (LNMO) is one of the most widely studied materials due to its large magnetodielectric properties and high Curie temperature T_c_~280 K [3,4,5]. For these reasons, LNMO has been considered as a promising candidate for the development of magnetoelectronic and spintronic devices and thus, has attracted much attention lately [6,7]. LNMO has a double perovskite structure (A_2_BB’O_6_), where Ni and Mn ions ideally occupy alternatively the B site positions giving place to a rocksalt-type lattice [5]. However, in thin film form, as occurs in other double perovskite systems, full B-site Ni-Mn cationic ordering is difficult to achieve [8,9,10,11]. In particular, in LNMO system, phase segregation often occurs and the coexistence of two structural phases is detected at room temperature [12,13], leading to the observation of two Curie temperatures [14].

To improve the magnetic properties of LNMO samples post-growth annealing processes are commonly employed [15,16,17]. Nonetheless, recent studies report the formation of the NiO phase impurities in LNMO thin films grown by MBE technique with the post-growth annealing, with apparently defect-free growth [18,19]. Detailed cross-sectional high-angle annular dark-field studies revealed an inverted “pyramid-like” shape morphology of the NiO precipitate, which progresses from a 2–3 nm base to a ∼10 nm wide mouth at the film surface [19]. The coexistence of double perovskite and NiO secondary phase is supported by first principles modeling of growth in oxygen deficient conditions with expected dissolution of inclusions during annealing [18]. Nanometer size nickel-oxide precipitates were also detected during the growth of LaNiO_3_-LaAlO_3_ superlattices on SrTiO_3_ substrate, changing their electronic properties [20]. Similar phase segregation processes are also observed in other manganite systems with cationic deficiency, strongly affecting magnetic and transport properties of the materials [21].

In this work, we report on the spontaneous formation of nickel-oxide nanocuboids at the surface of La_2_Ni_1−x_Mn_1+x_O_6_ thin films, deposited by RF magnetron sputtering technique. Annealing treatments in oxygen at high temperature (in-situ or ex-situ) after thin film growth promote the spontaneous formation of NiO nanocuboids giving place to the formation of a nanostructured surface. The evolution of the structural and magnetic properties of the films as a function of the annealing temperatures has also been studied. As expected, the post-annealing processes improve magnetic properties as well as structural properties, while modifying significantly the local transport properties at the film surface.

## 2. Materials and Methods

La_2_Ni_1−x_Mn_1+x_O_6_ (LNMO) thin films were fabricated by RF magnetron sputtering under a total oxygen pressure of 19 Pa at high temperatures (900 °C) on top of SrTiO_3_ (001) substrates using a stoichiometric La_2_NiMnO_6_ target. The mini-MAK from US-INC magnetron was 33 mm (1.3 inch) in diameter. The RF power used during thin film deposition was 40 W. The background pressure of the home-made sputtering chamber is in order of ~10^−5^ Pa. Electron probe microanalysis revealed Ni-deficiency (x~0.47) in the films [11]. Before deposition, substrates were cleaned in an ultrasonic bath with Milli-Q water and then annealed at 1000 °C in air for 2 h to obtain a clean and smooth TiO_2_ terminated surface [22]. Samples with thicknesses, t, of 40 nm < t < 50 nm, as determined by X-ray reflectivity, were prepared. After film deposition, as-grown samples were annealed (in-situ or ex-situ) for 1 h under high oxygen partial pressure (PO_2_~5 × 10^4^ Pa) to optimize the magnetic properties and oxygen stoichiometry. The evolution of the physical and structural properties was studied after successive annealing processes at 800 °C (Annealed-800 °C) and 900 °C (Annealed-900 °C) for 1 h of the same LNMO thin film.

The surface morphology of the LNMO film was characterized by scanning electron microscopy (SEM, QUANTA FEI 200 FEG-ESEM) and atomic force microscopy (AFM, MFP-3D AFM Asylum Research, Gole, CA, USA) in tapping mode. Structural characterization was made by means of X-ray diffraction (XRD) and reflectivity techniques (X’Pert MRD-Panalytical and a Siemens D5000, Malvern Panalytical, Malvern, UK). Reciprocal space mappings (RSM) were recorded using a Bruker-AXS General Detector Diffraction System (GADDS) model D8 Advance with a 2D detector. Energy dispersive X-ray analysis (EDX) has been performed on a FEI Magellan 400 L XHR SEM using an X-Max Ultim Extreme EDX detector (Oxford Instruments, Abingdon, UK). Transmission electron microscope (TEM) measurements were made by means of a field emission gun FEI Tecnai F20 microscope at 200 kV with a point-to-point resolution of 0.19 nm. Magnetization measurements were done using a superconducting quantum interference device (SQUID, Quantum Design, San Diego, CA, USA). The local electrical response of the films surface was explored by conductive atomic force microscopy (C-AFM), measurements were performed employing a MFP-3D microscope from Asylum Research with an Optimized Resistance Conductance Amplifier (ORCA) module. Diamond doped coated probes (DDESP-FM-V2 from Bruker, Billerica, MA, USA) were used in both the current maps and the I-V curves to ensure the stability of the tip during data acquisition. Moreover, these measures were carried out in a closed chamber with nitrogen atmosphere with the aim of reducing the ambient humidity and, therefore, avoid possible anodic oxidation effects.

## 3. Results and Discussion

**Figure 1:**
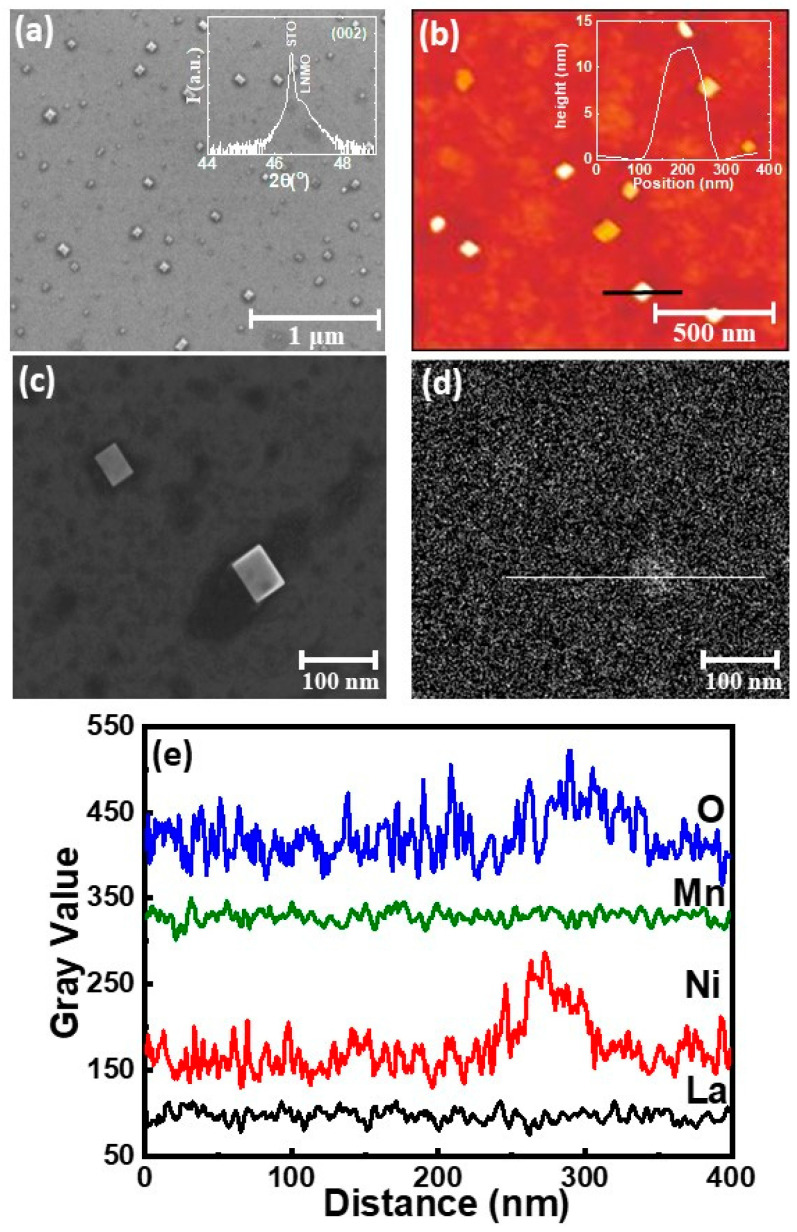
(**a**) SEM micrographs of LNMO-900 °C with in-situ annealing (thickness, t = 42 nm). Inset shows the *θ*/2*θ* XRD pattern around 002 bragg peak of the substrate. (**b**) AFM image of LNMO-900 °C film with the height profile of one nanocuboid in the inset. (**c**,**d**) Small area SEM and EDX map at energy of E = 15 eV with the corresponding profile spectra of different elements by X-ray spectroscopy analysis in (**e**).

As evidenced in Figure 1, the surface morphology of a LNMO film, after usual annealing in-situ process at 900 °C, reveals the existence of a nanostructured surface. The SEM image (Figure 1a) detects uniformly distributed nanocuboids throughout the film surface, with nearly square-based, sub-100 nm in size. AFM topography in Figure 1b confirms the presence of nanocuboids, distributed on top of a flat LNMO surface with low roughness (rms~0.3 nm). The estimated occupation of the surface is below 15%. The distribution of nanocuboids size is well described by the log-normal function with the most probable size of around S_0_~58 nm and the distribution width of σ~0.23 (see Figure A4 in Appendix B). The inset of Figure 1b reveals a nanocuboid height of ~12 nm. An interesting observation is that nanocuboids’ edges are mainly oriented along the [100] direction of the substrate (note that SEM and AFM images are rotated around 45° respect to the substrate edge during observation).

**Figure A1:**
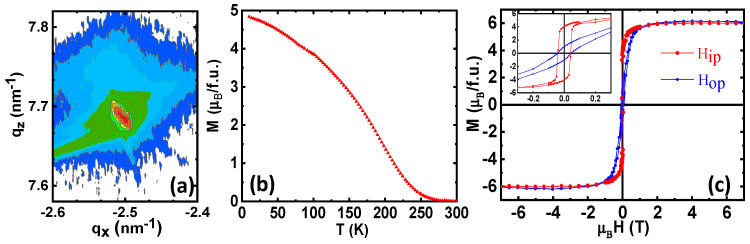
(**a**) RSM around (103) reflection of LNMO900 °C thin film (thickness, t = 42 nm) with in-situ annealing temperature at 900 °C (**b**) In-plane magnetization as a function of temperature under an applied field of µ_0_H = 0.5 T. (**c**) In-plane and out-of-plane hysteresis loops M(H) at 10. Inset shows the low field region in detail.

**Figure A2:**
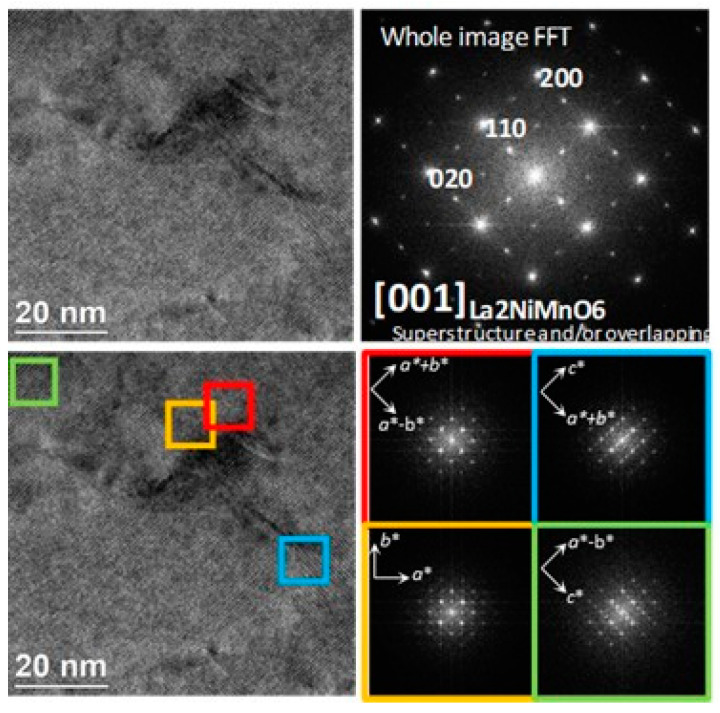
A plan-view HRTEM image of LNMO900 °C thin film. Fourier transform (FT) shows the coexistence of nanodomains with different orientations.

A deeper insight in the films’ structure was obtained by X-ray diffraction and reflectivity techniques. For simplicity and comparison between the film and the substrate, the LNMO lattice parameter is considered in pseudocubic notation (a_bulk_ = 3.876 Å) [23,24]. LNMO films on top of STO (a_STO_ = 3.905 Å) substrates grow under small tensile strain (a_LNMO_-a_STO_)/a_STO_ ×100% = −0.74%. The in-plane lattice parameter of the film determined from the reciprocal space maps, around (103)_STO_ reflection, perfectly matches with that of the STO substrate (see Figure A1a in Appendix A). The high resolution *θ*/2*θ* X-ray diffraction allows identifying the LNMO peak in the proximity of the intense substrate one (see inset of Figure 1a) with an out-of-plane lattice parameter of a_⊥_ = 3.877 ± 0.003 Å. The estimated LNMO unit cell volume (~59.12 Å^3^) is very similar to the unit cell volume of Ni-deficient bulk counterpart (LaNi_0.25_Mn_0.75_O_3+δ_) [12]. A planar view high resolution TEM (HRTEM) images supports XRD measurements, with a coexistence of domains with different orientations (Figure A2 in Appendix A).

Information regarding the chemical composition of the nanometric cuboids was obtained from energy dispersive x-ray (EDX) analysis. A small area SEM image of a single nanocuboid and the corresponding EDX map obtained at 15 KeV are shown in Figure 1c,d. The profile spectra of constituent elements (La L_α1_, Ni, Mn and O K_α1_) in a single nanocuboid are shown in Figure 1e, indicating the presence of nickel and oxygen. The presence of La and Mn have been excluded due to the very low detection percentage (no difference observed along the baseline in Figure 1d). Therefore, semi-quantitative EDX analysis indicate that the nanometric cuboids formed on top of the LNMO films surface are Ni-rich phase, most probably NiOx phases, in agreement with recent reports of the formation of NiOx segregates in LNMO double perovskites [18,19]. In those works, it is proposed that oxygen deficiency in LNMO films could favors the formation of antisite defects [23] and NiOx phase segregations [18,19].

**Figure 2:**
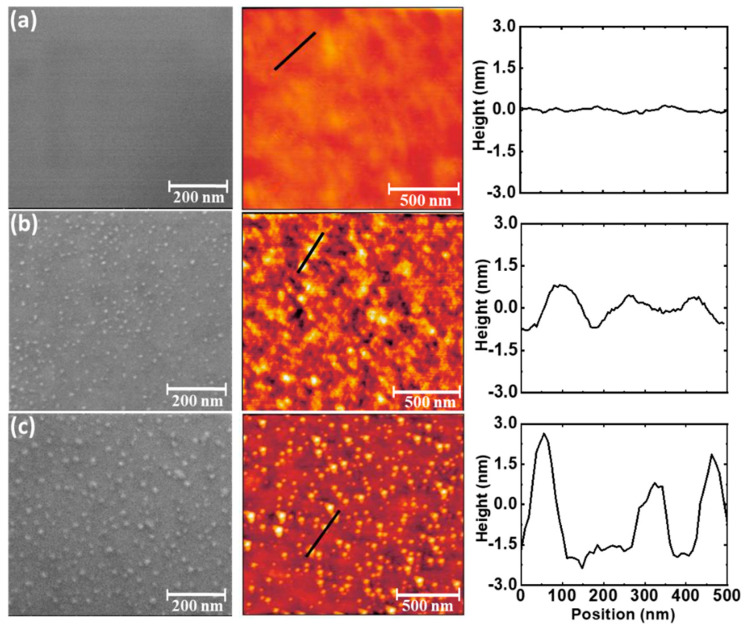
SEM micrographs (**left**-hand image) and AFM images (**right**-hand image) with the typical line profile of the same LNMO thin film (thickness, t = 48 nm) after different annealing processes: (**a**) as-grown film (no-annealing process), (**b**) annealing at 800 °C and (**c**) annealing at 900 °C.

To gain a deeper insight into the formation of NiOx nanocuboids, successive annealing processes at different temperatures are applied ex-situ to a non-annealed as-grown film. The evolution of the physical and structural properties was followed on the same LNMO thin film after each annealing process and the changes on the morphology are presented in Figure 2a–c. SEM (left-hand image) and AFM (right-hand image) micrographs in Figure 2a of the as-grown LNMO film without annealing process show a flat surface with an average roughness of rms~0.3 nm. In this case, no signals of segregation were detected.

However, after an ex-situ annealing at 800 °C, segregated nanostructures appears distributed through the whole surface as shown in Figure 2b. SEM micrographs revealed the presence of uniformly distributed small nanoparticles. The corresponding AFM confirms the presence of these nanoparticles, reflected in a higher value of global surface roughness (rms~0.45 nm). Log-normal distribution indicates the most probable nanoparticle size of around S_0_~13.5 nm with the distribution width of σ~0.13 (see Figure A5a in Appendix B). However, the mean-size of nanostructures remains rather small and with a height of few nanometers ~2 nm (see the line profile in Figure 2b).

Performing further ex-situ annealing process at higher temperatures (900 °C) on the very same LNMO film resulted in an enhancement of the growth of the nanoparticles. Nanoparticles became larger in-size as shown in Figure 2c). Log-normal distribution reveals the most probable nanoparticle size of S_0_~18.5 nm with the distribution width of σ~0.17 (see Figure A5b in Appendix B). The average nanoparticle height increases to around ~5 nm which is also reflected in an increase of the roughness (rms~0.7 nm). Although the density of nanoparticles appears to decrease by increasing the annealing temperature (from 5.34 × 10^−4^ nanoparticles/nm^2^ for Annealed-800 °C, to 3.70 × 10^−4^ nanoparticles/nm^2^ for Annealed-900 °C) the average volume of NiO_x_ at the surface has been estimated to increase around three times (see Table A2 in Appendix B), thus confirming that annealing process enhances the segregation.

**Figure 3:**
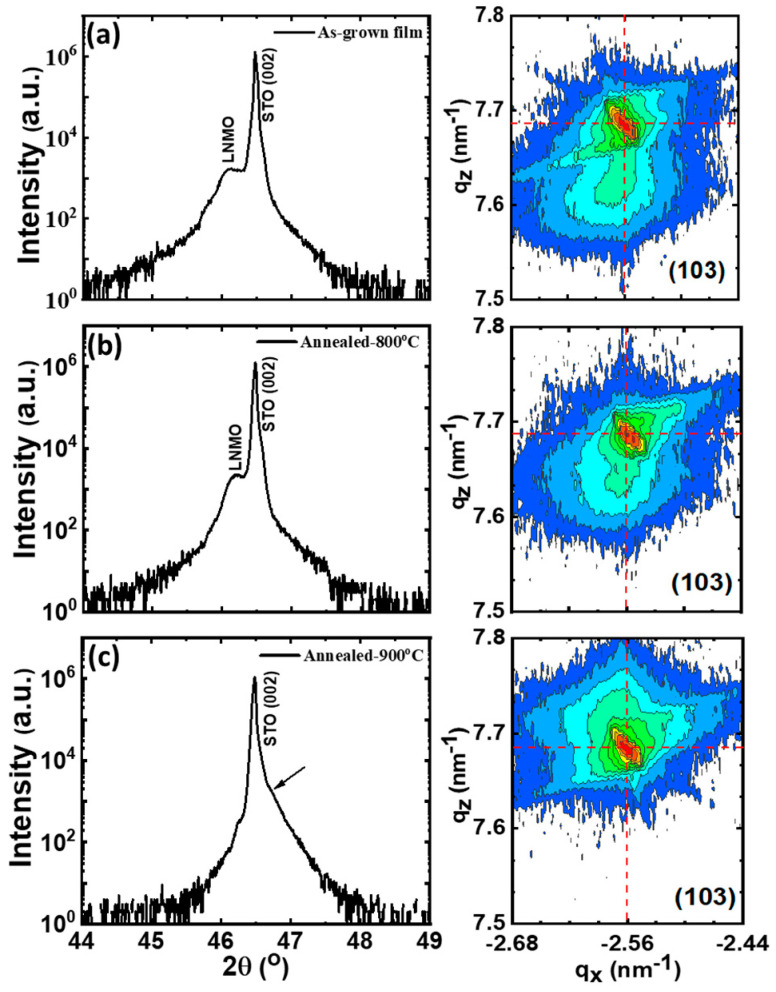
High-resolution *θ*/2*θ* XRD scans of the (002) reflections (**left**) and RSM around (103) reflection (**right**) of the same LNMO thin film (thickness, t = 48 nm) after different annealing processes: (**a**) as-grown film, (**b**) annealing at 800 °C and (**c**) annealing at 900 °C. The arrow indicates position of LNMO peak and the red dotted line indicates the position of the STO peak.

The effect of different annealing processes on the structural properties of the same LNMO thin film has been also followed by high resolution XRD scan of the (002) reflection and the asymmetric RSM around (103) reflection (Figure 3). In Figure 3a, a shoulder could be distinguished in the proximity of the prominent substrate (0 0 2) peak, which can be associated to the LNMO peak with an out-of-plane parameter of a_⊥_ = 3.939 ± 0.002 Å. At the same time, RSM around (103) reflection of the same film, indicates the position of the LNMO film just below the substrate peak (right-hand image in Figure 3a), confirming that the as-grown film is fully strained with the in-plane parameter that mimics the lattice parameter of underlying substrate (a_STO_ = 3.905 Å).

**Figure 4:**
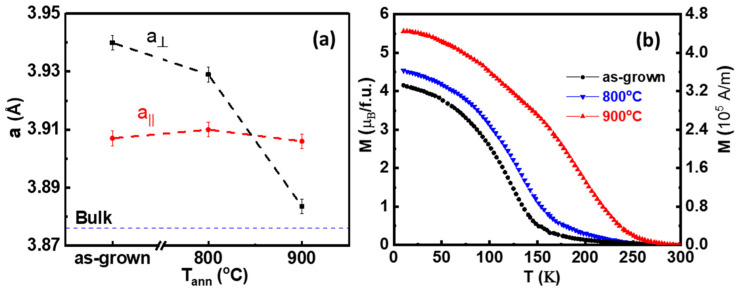
(**a**) Variation of in-plane (red dashed line) and out-of-plane (black dashed line) lattice parameters of LNMO film after different ex-situ annealing processes. The blue dashed line represents the bulk counterpart value [23] and (**b**) corresponding temperature dependence of in-plane magnetization at µ_0_H = 0.5 T.

The first annealing process at 800 °C (Figure 3b) does not significantly change the structural parameters. From *θ*/2*θ* XRD scans (left-hand image in Figure 3b), the out-of-plane lattice parameter is found to decrease to a_⊥_ = 3.929 ± 0.002 Å, remaining elongated respect to the corresponding pseudocubic perovskite lattice parameter of bulk LNMO (a_bulk_ = 3.876 Å) [23]. However, under annealing process at 900 °C in Figure 3c, the shoulder in θ/2θ XRD scan disappears completely and only the substrate peak can be clearly observed. Nevertheless, the RSM around (103) reflection indicates that the LNMO film peak is just above the substrate peak corresponding to a slight asymmetry detected in θ/2θ XRD scan (see arrow in left-hand Figure 3c). While the in-plane lattice parameter still remains constant (a_ǁ_ = 3.905 ± 0.005 Å), the out-of-plane lattice parameter further decreases to a_⊥_ = 3.883 ± 0.001 Å. The variation of the in-plane and the out-of-plane cell parameters in pseudo-cubic notation after different annealing processes are depicted in Figure 4a. The in-plane lattice parameters (red dashed line in Figure 4a) match the SrTiO_3_ lattice parameter indicating that the film remains strained even after various annealing processes. On the other hand, the out-of-plane lattice parameter (black dashed line in Figure 4a) decreases progressively towards the LNMO bulk value (blue dashed line).

It is worth to note that non-annealed as-grown film has a larger out-of-plane lattice parameter than expected and consequentially larger unit cell volume (~60.06 Å^3^). This larger unit cell usually denotes some oxygen deficiency and the presence of Mn^3+^ [25]. In fact, as can be deduced from Figure 4a, the shrinkage of the out-of-plane lattice parameter while in-plane parameters remain unchanged results in a reduction of the unit cell volume after successive annealing, which is consistent with oxygen incorporation during the annealing processes and the formation of Mn^4+^, as previously observed in similar manganite systems [25,26,27]. Indeed, as-grown non-annealed film exhibit clearly depressed values of Curie temperature (black curve in Figure 4b while Tc is enhanced in annealed films (blue and red curves), in agreement with previous observations in the LNMO system [15]. It should be noted that we cannot exclude the presence of NiOx segregations inside the film matrix and that the presence of NiO secondary phase may also contribute to the increment of the out-of-plane lattice parameter in as-grown samples, besides oxygen deficiency. These results agree with previous studies showing that annealing processes in oxygen rich atmosphere promote an increase of the Curie temperature in La_2_NiMnO_6_ films and other double perovskites [15,16]. Furthermore, it has been shown that post-growth annealing treatments are also effective to reduce the number of antisite defects [17] and to dissolve NiO segregations, thus contributing to increase the saturation magnetization [18]. However, in our case, in spite of a clear improvement of the ferromagnetic properties after post-growth annealing (ex-situ or in-situ, see also Figure A1 in Appendix A), nanometric nickel-oxide segregations are still present at the LNMO surface.

It is worth mentioning that Curie temperatures found in our samples although significantly improved, are still lower than that found in the bulk counterpart even after oxygen annealing at the highest temperature (900 °C). This could be attributed to the non-homogeneity of the sample detected by HRTEM (see Figure A2 in Appendix A), that causes the formation of NiO secondary phases (as detected from EDX measurements) and cationic vacancies, giving rise to octahedral distortions in the film, and thus affecting their ferromagnetic properties [28,29]. Moreover, as Ni agglomeration at the surface is larger after annealing process it is sound to assume that the Ni deficiency inside the film increases that, as a consequence, could further affect the ferromagnetic transition, as T_c_ is known to decrease when reducing the Ni content [3,12]. In conclusion, the strong structural strain exerted by the substrate on thin layers clamped to it allows only the contraction of the out-of-plane cell parameter. Thus, high-temperature annealing in oxygen atmosphere promotes a vertical cationic migration (the entry of oxygen ions as well as nickel migration towards surface), allowing the formation of nickel-oxide nanoparticles at film surface.

**Figure 5:**
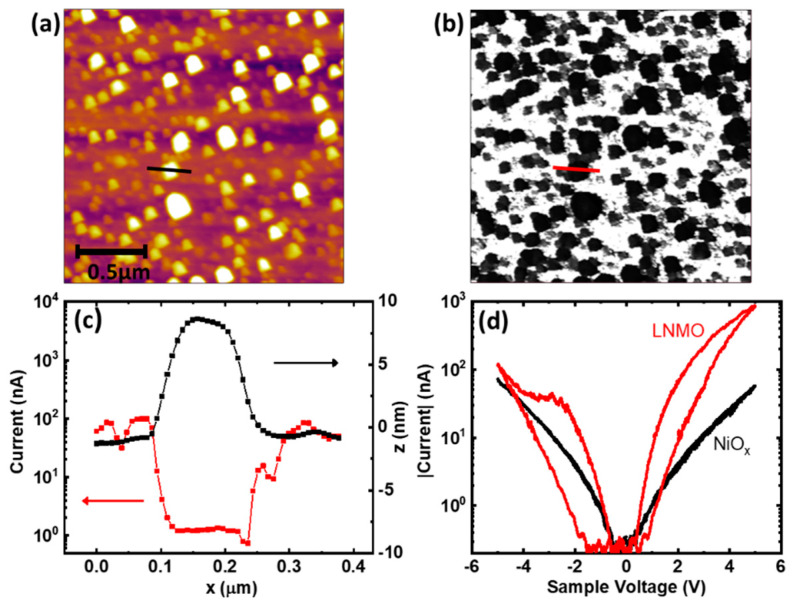
Local electric properties of the LNMO films with NiO_x_ nanoparticles at the surface measured by C-AFM. (**a**) Topography image of a 2 × 2 μm^2^ area of the film. (**b**) Simultaneous current map of the same area taken (applying 1.5 V), where the less conductive parts can be related with the particles observed in the topography. (**c**) Current (red) and height (black) profiles of a NiO_x_ nanoparticle and the surrounding LNMO film marked in (**a**,**b**). (**d**) I-V curves on the LNMO film (red) and on a particle (black), respectively.

Additionally, the presence of nickel-oxide nanoparticles at the surface of LNMO films may strongly influence local electrical properties. To analyze the electronic properties of the different phases present at the LNMO film surface, conductive atomic force microscopy measurements were performed in films prepared after an in-situ annealing at 900 °C. Figure 5a shows topographic image in a 2 × 2 μm^2^ area of a LNMO film with NiO_x_ nanoparticles formed on its surface, while Figure 5b depict its simultaneous acquired current map (applying 1.5 V).

In the topography image, NiO_x_ nanoparticles can be identified as the parts of the surface with higher height (bright circles) while in the current map a conductive matrix (white background) is disrupted by less conductive circles (black). Comparing both images allows identifying NiO_x_ nanoparticles as the less conductive parts of the current map, i.e., NiO_x_ particles are more resistive than the LNMO film. This correlation can be better appreciated in Figure 5c, where the profiles of the topography (black) and the current (red) of the lines (see Figure 5a,b) are depicted. In Figure 5c, it can be clearly observed that, in the higher height (z) region, which corresponds to a NiO_x_ particle, the current drops to ≈1 nA, while in the flat surrounding regions, where the LNMO film is directly measured, the current is almost two orders of magnitude higher (≈100 nA).

Furthermore, the response of the LNMO film and the NiO_x_ particles to voltage cycling is clearly different, as evidenced in Figure 5d. Voltage cycles taken directly on the LNMO surface (red curve) show a hysteretic behavior pinned at 0 V characteristic of a resistive switching behavior. At the same time, it is also observed that the application of a negative voltage (−5 V) locally increases the resistance of the film, switching the affected area to the so-called high resistance state (HRS). This HRS can be reverted back by the application of a positive voltage (+5 V) which returns the affected area of the film back into a more conductive state, i.e., the low resistance state (LRS).

**Figure A3:**
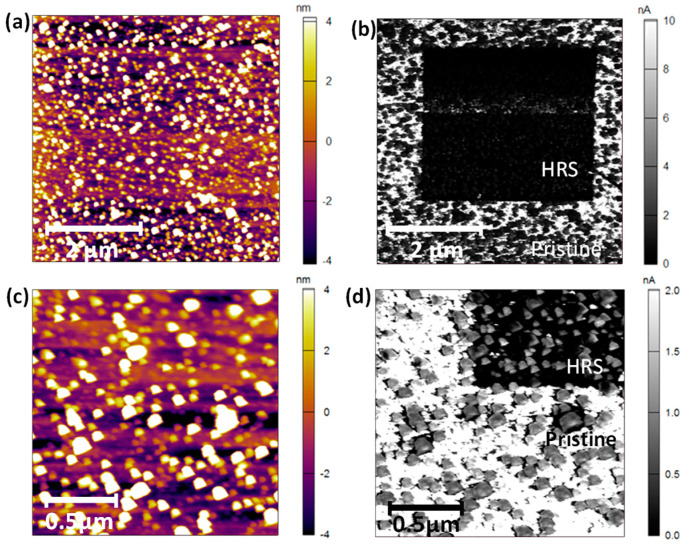
Switching of a 3 × 3 μm^2^ area of a LNMO film into the HRS. (**a**) Topography and (**b**) current map images of a 5 × 5 μm^2^ area, where the central 3 × 3 μm^2^ has been previously scanned at −4 V of sample Voltage. The current map shows a switching to the HRS of the LNMO film in the scanned area, which does not induce any changes in the topography. (**c**) Topography and (**d**) Current map images of a detailed area (2 × 2 μm^2^) showing the lack of switching of NiOx particles that are more resistive than the film in the pristine state but more conductive than the film in the HRS.

**Figure A4:**
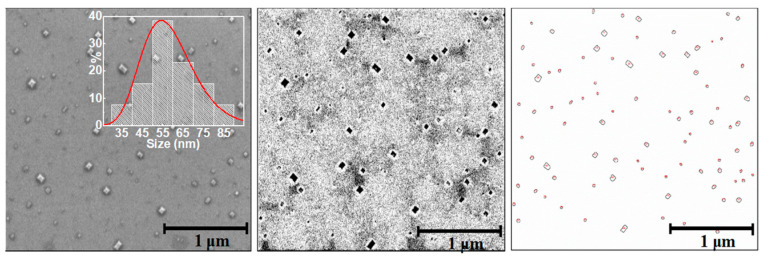
SEM micrograph with a magnification of 50,000× of LNMO-900 °C thin film (thickness, t = 42 nm, in-situ annealing at 900 °C). Filtered image of its corresponding SEM image (**medium**) and automated grains counting with Image J software (**right**).

**Figure A5:**
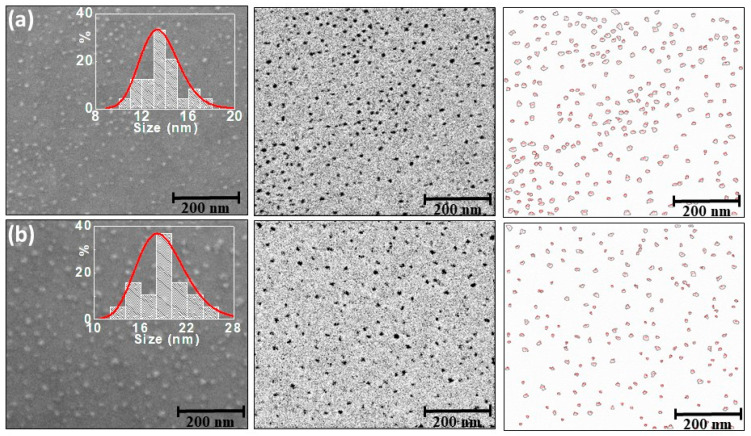
SEM micrographs with a magnification of 200,000× of the same LNMO thin film (thickness, t = 48 nm) after different ex-situ annealing temperatures: (**a**) Anneal-800 °C and (**b**) Anneal-900 °C. Filtered image of its corresponding SEM image (**medium**) and automated grains counting with Image J software (**right**).

It is important to mention that both HRS and LRS are of a non-volatile nature and can be switched between them multiple times, stating the base for a bipolar resistive switching behavior suitable for the implementation of memory devices. A totally different behavior is observed sweeping the voltage on the top of a particle (black line). In this case, a non-hysteretic symmetrical curve is obtained, where the application of positive or negative voltage does not have any influence in the resistivity properties of the particles. The absence of resistive switching in the particles can be also visualized in Figure A3 (Appendix A). After switching an area to the HRS, the LNMO current is heavily reduced while the particles included in the area maintain their initial current, thus showing more conductivity than the high resistive state. Thus, our results show that nanostructured LNMO films may be switched from a system of poor conductive particles onto a conductive matrix to the opposite case, with conductive particles onto an insulating matrix, thus behaving as a model to study interfacial local transport.

## 4. Conclusions

In conclusion, we report on the controlled formation of nickel-oxide nanocuboids at the surface of La_2_Ni_1−x_Mn_1+x_O_6_ (x~0.47) thin films deposited by the RF magnetron sputtering technique. It is shown that the LNMO functional properties are highly influenced by the post-growth annealing process. Even LNMO films remains strained after various consecutive annealing processes, annealing treatments in oxygen atmosphere at high temperature allow a partial structural relaxation reflected in a reduction of the unit cell volume towards the LNMO bulk value. This structural relaxation, detected through a reduction of the out-of-plane cell parameter, allows some kind of vertical cationic migration and promotes an improvement of the ferromagnetic properties that affects both the Curie temperature and the saturation magnetization of the films. On the other hand, oxygen annealing processes promote Ni segregation towards the surface of the films where the formation of NiO_x_ nanoparticles is detected. The presence of NiO_x_ nanocuboids significantly changes the local surface transport properties, allowing one to obtain a composite surface in which areas exhibiting a resistive switching behavior alternate with other insulating behaviors.

## Data Availability

The data that support the findings of this study are available on request from the corresponding authors.

## Appendix A

Figure A1 shows structural and magnetic properties of La_2_Ni_0.53_Mn_1.47_O_6_ thin film (thickness, t = 42 nm) with in-situ annealing temperature at 900 °C for 1 h under 5 10^4^ Pa O_2_ (growth at 900 °C under PO_2_~19 Pa). The reciprocal space map, around (103)_STO_ reflection, indicates strained film as discussed above (Figure A1a). The T_c_ value found in our samples is slightly lower compared to the bulk value, as expected for nickel deficient samples [3,12] (Figure A1b). In-plane and out-of-plane hysteresis loops M(H) at 10 K (Figure A1c) exhibit the expected hysteretic behavior with similar coercive field, H_c_~400 Oe (see corresponding inset). On the other side, a saturation magnetization of (M_s_ ≈ 5.8 µB/f.u) is found to be somewhat larger than the expected theoretical value (∼5 μB/f.u.) for fully ordered stoichiometric LNMO, as discussed previously [11]. More details about the ferromagnetic nature in LNMO films with similar magnetic behavior, including X-ray magnetic circular dichroism, can be found in [11].

Figure A2 shows a plan-view in high resolution TEM (HRTEM) images, acquired in order to obtain information about the microstructure and crystallographic features of a LNMO film with in-situ annealed-900 °C process. The sample was cut parallel to the sample surface (perpendicular to the (00l) planes). HRTEM images show a non-homogeneous surface with the coexistence of domains with different orientations. The Fourier transform (FT) of selected areas of the image were computed in order to assess the periodicity of the crystal lattice. The FT of a large area was marked by squares, which shows different spots corresponding to the enlargement of the LNMO lattice with respect to STO (LNMO cell parameters are of the type a ≈ b ≈ √2a_p_, c ≈ 2a_p_ where a_p_ is the primitive perovskite cell parameter). These spots can be indexed as (0 k/2 l/2) (k and l odd), (0 k/2 l/) (k odd), and (0 k l/2) (l odd) and indicate different orientations of the LNMO cell in the film.

The Resistive switching characteristics (or their absence), depicted in Figure 5d of the main text, has also been probed on large surface areas to assess its reproducibility. Figure A3a,b depicts the topography and current images of a 5 × 5 μm^2^ area. In the center of this area a 3 × 3 μm^2^ square has been previously scanned at −4 V. As a consequence of this initial scan, the area has been switched into the HRS without harming the surface, as can be noticed in the topography image. Figure A3c,d show a detail picture (2 × 2 μm^2^) of the previous image. Adjusting the scale of the current map it can be observed that, inside the switched area, the particles are more conductive than the LNMO film in the HRS, while in the pristine state the particles are more resistive. The lack of resistance change after the biased scan supports the I-V curves presented in the main text and confirms the absence of RS behavior in the NiO_x_ particles.

## Appendix B

The size of nanocuboids was determined from SEM micrographs using image analysis (ImageJ software [30]) averaging measurements of a large number of particles at different regions of the film surface. The distribution of nanoparticle size is well described by the log-normal function $f\left(S\right)=\frac{1}{\sqrt{2\pi }\sigma S}\exp\left[-\frac{ln^{2}\left(\frac{S}{S_{0}}\right)}{2\sigma^{2}}\right],\;$where the fitting parameters *S*_0_ and *σ* are the most probable nanoparticle size and the width of the distribution, respectively. The average nanoparticle size S_M_ is determined from the most probable nanoparticle size (*S_M_* = *S*_0_ exp(*σ*^2^/2)). Examples of the analysis performed for different thin films are shown in Figure A4 (in-situ annealed film) and Figure A5 (ex-situ annealed film). A summary of the obtained values for the surface density and average nanoparticle volume is presented in Table A1 and Table A2.

**Table A1:** Surface density, average nanoparticle volume, total volume of the nanoparticles (NiO_X_) per unit surface (1 µm^2^) and nanoparticle fraction volume of LNMO-900 °C thin film (determined from Figure A4).

Annealing T (In-Situ)	Density (NP/µm^2^)	NP Volume (nm^3^)	NPs Volume (nm^3^)/µm^2^	NP Fraction Volume
900 °C	~1.04 × 10^−1^	~4.2 × 10^4^	~4.4 × 10^5^	~0.011

**Table A2:** Surface density, average nanoparticle volume, total volume of the nanoparticles (NiO_X_) per unit surface (1 µm^2^) and nanoparticle fraction volume of the same LNMO thin film after different ex-situ annealing temperatures, determined from Figure A5.

Annealing T (Ex-Situ)	Density (NP/µm^2^)	NP Volume (nm^3^)	NPs Volume (nm^3^)/µm^2^	NP Fraction Volume
800 °C	~5.34 × 10^2^	~3.7 × 10^2^	~2.0 × 10^5^	~0.004
900 °C	~3.70 × 10^2^	~1.8 × 10^3^	~6.5 × 10^5^	~0.014
